# Targeting SETDB1 in cancer and immune regulation: Potential therapeutic strategies in cancer

**DOI:** 10.1002/kjm2.12933

**Published:** 2025-01-07

**Authors:** Bo‐Syong Pan, Cheng‐Yu Lin, Gilbert Aaron Lee, Hui‐Kuan Lin

**Affiliations:** ^1^ Department of Pathology Duke University Medical Center Durham North Carolina USA

**Keywords:** cancer, endogenous retrovirus silencing, immune checkpoint blockage, methyltransferase, SETDB1

## Abstract

SET domain bifurcated histone lysine methyltransferase 1 (SETDB1/ESET), a pivotal H3K9 methyltransferase, has been extensively studied since its discovery over two decades ago. SETDB1 plays critical roles in immune regulation, including B cell maturation, T‐cell activity modulation, and endogenous retrovirus (ERV) silencing. While essential for normal immune cell function, SETDB1 overexpression in cancer cells disrupts immune responses by suppressing tumor immunogenicity and facilitating immune evasion. This is achieved through the repression of anti‐tumor immune cell production, ERV silencing, and interference with the type I interferon pathway leading to inhibiting immune checkpoint blockade (ICB) efficacy. Beyond its immunological implications, SETDB1 overexpression fosters tumor growth and metastasis via transcriptional silencing of tumor suppressor genes through histone regulation and activating oncogenic signaling by non‐histone regulation. These multifaceted roles make SETDB1 an attractive epigenetic target for novel cancer therapies. This review explores SETDB1's dual function in immune regulation and tumor progression, emphasizing its potential in the development of innovative cancer treatments targeting epigenetic dysregulation and oncogenic signaling.

## 1 INTRODUCTION

SET domain bifurcated 1 (SETDB1) is a histone H3K9 methyltransferase critical for gene silence involved in diverse cellular processes, including cell cycle regulation, apoptosis, differentiation, epithelial–mesenchymal transition (EMT), cell growth, and metabolism. By promoting heterochromatin formation, SETDB1 represses transcription in conjunction with co‐repressors, such as the human silencing hub (HUSH) complex,^1^ KRAB‐zinc finger proteins (KRAB‐ZFP),^2^ and KAP1/TRIM28.^3^ Beyond its established roles in cancer progression and proliferation, SETDB1 has recently been recognized as a key regulator of immune cell differentiation and function by affecting processes, such as B and T lymphocyte development, cytokine production, and activation of innate immune genes. These functions are mediated by histone H3K9 trimethylation, which silences gene promoters or suppresses endogenous retroviral (ERV) elements that could otherwise act as enhancers. This review highlights the involvement of SETDB1 in immune cell development, immune evasion, and innate immune responses within the context of cancer, exploring its dual functions in immune regulation and tumor progression while emphasizing its potential in developing innovative cancer treatments targeting epigenetic dysregulation and oncogenic signaling.

## 2 KMTs, THE SET DOMAIN FAMILY, AND SETDB1

Histone proteins, which form the structural core of nucleosomes, are subject to various post‐translational modifications, such as acetylation, phosphorylation, lactylaton, and methylation, all of which regulate gene expression.^4^ Among these, histone methylation plays a vital role in preserving heterochromatin organization and the transcriptional memory of cells. Histone methyltransferases (HMTs) catalyze the addition of methyl groups to lysine or arginine residues in histone tails, with lysine‐specific methyltransferases (KMTs) responsible for mono‐, di‐, and trimethylation of lysine residues. Dysregulation or mutations of KMTs are frequently associated with cancer and developmental disorders.^5^

**FIGURE 1:**
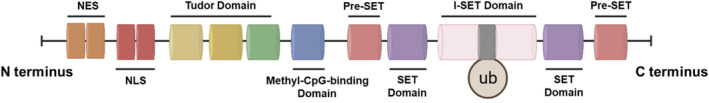
SETDB1 structure and functional domains. SETDB1 contains a conserved SET domain, a variable I‐SET insert, and a unique triple TUDOR domain (TTD) that regulates its interaction with nucleosome. NES, nuclear export signal; NLS, nuclear localization signal.

The SET domain, a defining feature of many KMTs, governs their catalytic activity. SET‐domain KMTs are categorized based on structural and sequence similarities, with the SUV39 family being particularly important for histone H3 lysine 9 (H3K9) methylation. SETDB1, a member of this family, mediates H3K9 di‐ and trimethylation in euchromatic regions, dynamically regulating chromatin structure and gene expression.^6^ Structurally, SETDB1 contains a conserved SET domain, a variable I‐SET insert, and a unique triple TUDOR domain (TTD) that regulates its interaction with nucleosomes. A closely related paralog, SETDB2, also methylates H3K9 in a tissue‐specific manner, demonstrating variations in SET domain sequences due to the I‐SET insert (Figure 1).

## 3 SETDB1 STRUCTURE AND FUNCTIONAL DOMAINS

1. N‐terminal domains
  ○ *TUDOR domain*: Recognizes chromatin marks like H3K9me1/2 and H3K14ac, promoting H3K9 trimethylation and mediating protein–protein interactions critical for transcriptional repression.
  ○ *Methyl‐DNA binding domain (MBD)*: Facilitates recognition of methylated DNA, particularly CpG islands, linking DNA methylation with histone modification to enable gene silencing.
2. C‐terminal SET domain
  ○ Comprising pre‐SET, SET, and post‐SET subdomains, this domain is essential for catalytic activity. The pre‐SET region stabilizes the structure via zinc‐coordinated cysteine motifs, while the post‐SET region aids in methyl group transfer by orienting the substrate.
3. Bifurcated SET domain
  ○ A unique 347‐amino‐acid insertion bifurcates the SET domain, potentially enabling distinct regulatory functions. Though not directly catalytic, this insertion can undergo post‐translational modifications, thus influencing enzymatic activity.
4. Mechanism of methyl transfer
  ○ SETDB1 catalyzes methyl transfer from S‐adenosylmethionine (SAM) to lysine residues via an SN2 mechanism. Tyrosine residues in the post‐SET domain stabilize the process by orienting substrates and co‐substrates.
5. Regulation and interaction
  ○ SETDB1 interacts with HDAC1/2 and KRAB‐ZFP‐KAP1 complexes to mediate transcriptional silencing, chromatin remodeling, and gene regulation.

## 4 PHYSIOLOGICAL ROLE OF SETDB1

SETDB1 is critical for H3K9 trimethylation‐mediated gene and retrotransposon silencing, with its activity regulated by ATF7IP, which promotes its nuclear retention and enhances its enzymatic functions.^7^ Recent findings reveal novel roles for SETDB1 beyond repression, such as co‐binding with CTCF and cohesin in domains involving SETDB1 and cohesin (DiSC), regions devoid of repressive histone marks, suggesting a role in maintaining cohesin complex integrity.^8^ Additionally, SETDB1 forms a repressive complex with KAP1 and KRAB zinc finger proteins (KRAB‐Zfps), silencing genes and ERVs while maintaining genomic stability.^9^ It also interacts with DNMT3s, particularly DNMT3A, in processes involving both dependent and independent DNA methylase activity,^10^ highlighting its role in DNA methylation maintenance and de novo epigenetic silencing, including at specific promoters like RASSF1A and FOXA2 in NSCLC cells.^11^ These findings underscore SETDB1's multifaceted roles in epigenetic regulation and genomic stability.

## 5 ROLE IN CANCER

**FIGURE 2:**
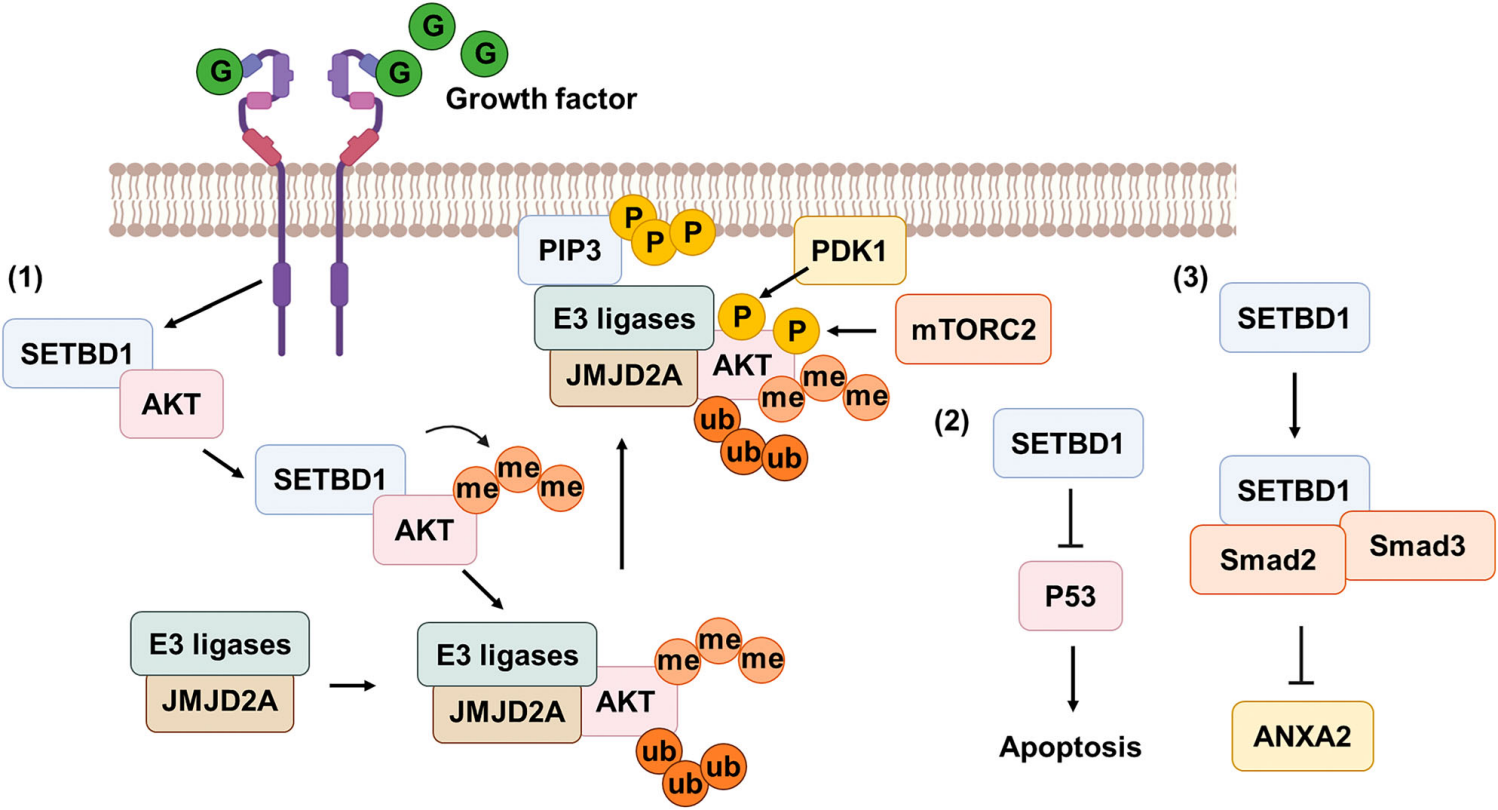
SETDB1 activates important cancer signaling pathways through non‐histone protein methylation and/or association. (1) SETDB1 interacts with Akt upon growth factor engagement to its receptor and triggers methylation of Akt at K64, which recruits JMJD2A along with E3 ligase to induce K63‐lined ubiquitination of Akt leading to Akt membrane recruitment and activation. (2) SETDB1 interacts with p53 and triggers p53 dimethylation at K370, leading to increasing recognition and degradation of p53 by MDM2 and prevents apoptosis. (3) SETDB1 associates with Smad2/3 complex upon TGF‐β treatment to repress ANXA2 to regulate metastasis.

SETDB1, a lysine methyltransferase, is pivotal in silencing tumor suppressor genes and plays a significant role in various cancer pathways.^12^, ^13^, ^14^ Overexpression of SETDB1 is associated with aggressive phenotypes in cancers, such as breast, colon, liver, lung, and myeloid leukemia, where its activity promotes rapid cell growth, proliferation, and immune evasion.^15^ For instance, SETDB1 knockdown induces apoptosis in acute myeloid leukemia (AML) cells through immune response pathways,^16^ underscoring its oncogenic role. Interestingly, SETDB1 exhibits cell‐type‐specific functions, acting as a suppressor of EMT in lung and breast cancers while facilitating metastasis in hepatocellular and gastric cancers.^17^, ^18^, ^19^ Mechanistically, SETDB1 influences cellular invasion and migration through interactions with key molecules like E‐cadherin, β‐catenin, ANXA2, Sp1, and Tiam1.^17^, ^19^ Beyond epigenetic silencing, SETDB1 directly activates oncogenic proteins, such as AKT activation through lysine methylation of AKT at K64, facilitating its ubiquitination, membrane recruitment, and activation by PDK1. SETDB1 also enhances AKT's activity via methylation on K140/142 residues at the PHD domain, promoting PIP3 binding and membrane translocation.^20^, ^21^ These findings highlight SETDB1 as a dual‐function molecule with both oncogenic and suppressive effects, making it a critical player in carcinogenesis and a potential therapeutic target (Figure 2).

## 6 SETDB1 IN B CELL DEVELOPMENT

SETDB1 is essential for B cell maturation by regulating epigenetic silencing of ERVs and retrotransposons. Collins et al. found that targeted SETDB1 deletion in pre‐B cells via Mb1‐CRE transgene results in a complete loss of B cell populations in bone marrow and spleen, linked to decreased H3K9 methylation and de‐repression of ERVs.^22^ These ERVs act as enhancers for non‐B cell lineage genes, disrupting B cell‐specific differentiation. Further studies revealed that SETDB1 silences retrotransposons like MLV, which, when upregulated, alter chromatin structure, activate the unfolded protein response (UPR), and induce apoptosis.^23^ Collectively, these findings highlight the critical role of SETDB1 in B cell development by repressing ERVs and retrotransposons to maintain lineage specificity and cell survival.

## 7 SETDB1 IN T CELL REGULATION AND DEVELOPMENT

SETDB1 plays a multifaceted role in T cell development, function, and lineage commitment by modulating gene expression through H3K9 methylation. Wakabayashi et al. demonstrated that SETDB1 and SUV39H1 suppress IL‐2 transcription in CD4^+^ T cells by cooperating with Smad2/3 to methylate the IL‐2 promoter.^24^ Similarly, SETDB1 represses IL‐17 expression in Th17 cells, as shown by Xiao et al., through its recruitment by OX40 and RelB to the IL‐17 locus,^25^ suggesting potential therapeutic applications in autoimmune diseases. In CD8^+^ T cells, SETDB1 prevents upregulation of FcγRIIB, which otherwise disrupts ERK signaling and T cell maturation, highlighting its importance in thymocyte development.^26^ Adoue et al. revealed that SETDB1 is crucial for naive CD4^+^ T cell differentiation, ensuring Th2 lineage stability and preventing reprogramming into Th1 cells by silencing ERVs near Th1 enhancers.^26^, ^27^ Overall, SETDB1 suppresses cytokine production, supports T cell maturation, and maintains lineage fidelity, underscoring its significance in adaptive immunity.

## 8 SETDB1 AND INNATE IMMUNE CELL FUNCTION

SETDB1 regulates innate immune cells including monocytes, macrophages, and dendritic cells by silencing genes that could lead to excessive immune activation. For example, it represses CD1a expression in monocytes and dendritic cells, limiting antigen presentation.^28^ In macrophages, SETDB1 inhibits pro‐inflammatory cytokines like IL‐6 and IL‐12 by methylating their promoters, thereby suppressing NF‐κB‐mediated transcription.^29^ Additionally, SETDB1 cooperates with KAP1 to silence ERVs, maintaining H3K9 methylation and preventing activation of RNA‐sensing pathways and innate immune genes.^30^ These roles establish SETDB1 as a central regulator of immune homeostasis and a potential therapeutic target for autoimmune and inflammatory disorders.

## 9 SETDB1 MODULATES LYMPHOCYTE ACTIVITY AND CYTOKINE EXPRESSION

SETDB1, commonly overexpressed in various cancers, plays a critical role in immune regulation and tumor progression. In breast cancer, SETDB1 represses IL‐6 expression and is linked to immune‐related genes, such as IL‐6 and HLA‐DPA1.^31^ Knockdown studies show elevated IL‐6 levels, emphasizing its immune‐modulatory functions.^29^ Similarly, in multiple myeloma, high SETDB1 expression is associated with poor prognosis, as it enhances NK cell activity while reducing T cell and dendritic cell infiltration.^32^ Furthermore, SETDB1's interaction with immune checkpoint genes and microsatellite instability^33^ highlights its broad impact on the immune environment and cancer development.

## 10 SETDB1 FACILITATES TUMOR IMMUNE ESCAPE

Recent studies have highlighted SETDB1 as a key player in tumor immune evasion and immune checkpoint therapy resistance. Griffin et al. found that targeting SETDB1 using CRISPR‐Cas9 in mouse models enhanced sensitivity to immune checkpoint therapies, with a marked decrease in tumor growth and activation of immune responses.^34^ SETDB1 depletion led to the activation of transposable elements (TEs) and triggered an interferon response, promoting NK and CD8^+^ T cell activation.^34^, ^35^ Further research by Zhang et al. identified another chromatin modifier, KDM5B, which is involved in the recruitment of SETDB1 to retroelements, enhancing immune evasion through silencing of these elements.^35^ In a lung cancer model, Hu et al. found that SETDB1 knockout enhanced tumor immunogenicity and interferon responses.^36^ Additionally, Tian et al. reported that SETDB1 regulates PD‐L1 expression in colorectal cancer through a complex pathway involving FOSB, miR‐22, and BATF3.^37^ These findings suggest that targeting SETDB1 or related pathways could improve the effectiveness of immunotherapies across various cancers.

## 11 SETDB1 DISRUPTS THE TYPE I INTERFERON RESPONSE

The type I interferon (IFN) response is a key antitumor mechanism.^38^ Recent studies suggest that SETDB1 suppresses this pathway through various mechanisms. Cuellar et al. identified SETDB1 as a critical factor in cell viability and survival in AML, with SETDB1 depletion leading to upregulation of type I IFN pathway genes and interferon‐stimulated genes (ISGs), such as IFIT1‐3 and MDA5.^16^ This was accompanied by the activation of retrotransposons, which induced the IFN response. However, SETDB1 depletion did not affect ISG‐related genes or innate immune sensors. Disruption of the dsRNA‐sensing pathway diminished the IFN response and increased cell survival in SETDB1‐depleted AML cells.^16^ In squamous‐cell carcinoma (SCC) and lung adenocarcinoma (ADC), high SETDB1 expression correlated with reduced innate immune response and increased RNA interference and chromatin modification gene expression.^39^ In contrast, low SETDB1 expression in melanoma (SKCM) and non‐small cell lung cancer (NSCLC) patients was associated with better radiotherapy outcomes, greater immune cell infiltration, and enhanced IFN response following SETDB1 depletion.^39^ These findings suggest that SETDB1 impedes immune responses, hindering T cell infiltration and contributing to immune evasion, with implications for improving cancer therapies by targeting SETDB1.

## 12 SETDB1 ACTIVITY DECREASES ICB EFFECTIVENESS

Immune checkpoint blockade (ICB) therapy is a revolutionized strategy by inducing immune responses against tumors. SETDB1 appears to drive immune therapy resistance by reducing its effectiveness. In 2021, Lin et al. identified SETDB1 as a critical histone modifier negatively regulating PD‐L1 in ovarian cancer, acting in concert with the TRIM28/KAP1 complex.^40^ This contradicts a previous study in colorectal cancer (CRC), where SETDB1 was shown to induce PD‐L1 and promote immune escape.^37^ However, TCGA data from ovarian cancer supported the CRC findings, showing a negative correlation between SETDB1 and CD8^+^ T cell infiltration.^40^ Mechanistically, SETDB1 knockout in ovarian cancer led to mitotic defects, micronuclei formation, and upregulation of PD‐L1 via the cGAS‐STING pathway. Later, Guo et al. confirmed that downregulating SETDB1 enhanced ICB response in ovarian cancer, showing that SETDB1 repression activated interferon signaling through both cGAS‐STING and RNA sensing pathways.^41^ This suggests a complex regulation of immune responses by SETDB1, although the specific factors driving differential activation of these pathways remain unclear.

## 13 TARGETING SETDB1 INHIBITION WITH SMALL MOLECULE DRUGS

The findings that SETDB1 plays a critical role in tumorigenesis and cancer progression, particularly in immune evasion and resistance to ICB therapies, place SETDB1 as a promising target for anti‐tumor therapies. However, research on SETDB1 inhibitors and associated crystal structures is still in its early stages, with limited development of SETDB1 antagonists. SETDB1 functions as an epigenetic modulator by adding methyl groups to histones, a process that occurs at the SET domain of SET‐domain family proteins.^42^ The donor methyl group of the co‐substrate S‐adenosylmethionine (SAM) interacts with the deprotonated nitrogen of the substrate lysine, forming a catalytic site made up of two distinct pockets, one for the substrate peptide and the one for the co‐substrate. This dual‐pocket structure offers two potential strategies for drug design: competitive binding to the substrate peptide or inhibition of the co‐substrate. Most of the effective SET domain inhibitors currently available, such as G9a inhibitor–UNC0638, compete with the substrate lysine residue. The structural specificity of SET‐domain proteins, including SETDB1, can be partially explained by the region between the N‐SET and C‐SET domains, although variations in the insert regions of different SET proteins complicate the prediction of selective inhibitors. This is evident in inhibitors like UNC0638, which specifically targets the H3K9 methyltransferases G9a and GLP, but not other H3K9 methyltransferases like SUV39H1 or SETDB1, and other methyltransferases like SETD7, SETD8, or SUV39H2. This highlights the complexity of designing inhibitors that are both potent and selective. While selective inhibitors have been reported for some H3K9 methyltransferases, such as SUV39H1, G9a, and GLP, much work remains to be done in developing selective and effective inhibitors for SETDB1 and other SET‐domain family members. Despite these challenges, SETDB1 remains an exciting therapeutic target, particularly in overcoming immune resistance mechanisms in cancer.^15^

## 14 BIX‐01294

Histone lysine methylation is a crucial epigenetic modification that regulates chromatin structure and gene expression.^43^ To explore its role and modulate its activity, previous research conducted a screen to identify inhibitors of histone lysine methyltransferases (HMTases), focusing on the G9a enzyme.^44^ From a chemical library of 125,000 compounds, seven hits were identified, with BIX‐01294 (a diazepin‐quinazolin‐amine derivative) standing out as a selective inhibitor of G9a. Unlike other inhibitors, BIX‐01294 does not compete with the cofactor SAM, but instead specifically targets G9a, reducing the methylation of H3K9 to H3K9me2 in vitro. In cellular assays, BIX‐01294 treatment in several cell lines caused a reversible reduction in bulk H3K9me2 levels, which were restored after the inhibitor was removed. Chromatin immunoprecipitation (ChIP) further confirmed that treatment with BIX‐01294 in mouse embryonic stem (ES) cells and fibroblasts resulted in a transient decrease in H3K9me2 at promoter regions of G9a target genes.^45^

BIX‐01294 is highly selective for G9a, with an IC50 of 1.7 μM, and shows only minor activity against the closely related G9a‐like protein (GLP) at higher concentrations. This selectivity makes BIX‐01294 a useful tool for studying G9a‐mediated epigenetic regulation. Importantly, BIX‐01294's ability to reduce H3K9me2 levels at G9a target genes in living cells suggests its potential for transient modulation of histone marks, making it a promising candidate for further research and therapeutic development. Its molecular structure, in contrast to other natural product‐derived HMTase inhibitors, allows for optimization into more potent inhibitors. Future studies, including the crystallization of BIX‐01294 bound to the G9a‐SAM complex, will be key for understanding its binding mechanism and improving its efficacy.

Additionally, previous research presented the crystal structure of the GLP SET domain in complex with BIX‐01294 and S‐adenosyl‐L‐homocysteine (SAH). The inhibitor binds to the substrate peptide groove in a position similar to histone H3 residues N‐terminal to the target lysine. BIX‐01294 mimics the conformation of histone H3 Lys4 to Arg8, stabilized by specific interactions with residues unique to both G9a and GLP. This structural insight provides a foundation for understanding the selective inhibition of G9a and GLP by BIX‐01294 and informs future efforts to design more effective inhibitors targeting HMTases.^46^

## 15 UNC0638

UNC0638, a selective inhibitor of the H3K9 methyltransferase G9a, has emerged as a key tool for studying epigenetic regulation in both hematopoiesis and embryonic development.^47^ This compound specifically targets the modification of histone H3K9, a critical epigenetic mark involved in gene silencing. Recent studies have demonstrated the significant impact of UNC0638 on modifying epigenetic landscapes and influencing gene expression, both in the context of AML and somatic cell nuclear transfer (SCNT) embryos.^47^

In the case of AML, where the HOXA gene cluster is frequently deregulated, UNC0638 has been shown to exhibit cytotoxic effects on myeloid leukemia cells by altering H3K9 methylation.^48^ The inhibition of H3K9 methyltransferases with UNC0638 results in the reactivation of genes that are typically suppressed by these epigenetic marks, including key pro‐leukemic genes, such as Hoxa9. Notably, studies have found that high SETDB1 expression, known to deposit H3K9 trimethylation, can suppress AML progression by promoting differentiation of leukemia cells.^47^ Treatment with UNC0638 in AML models reveals a potential therapeutic strategy by disrupting the epigenetic repression of these critical genes, leading to a suppression of leukemogenesis.^49^

Similarly, in the context of SCNT, where reprogramming of somatic cells to a pluripotent state is required for successful embryo development, UNC0638 has shown promise in correcting abnormal H3K9 methylation patterns observed in SCNT embryos. In goat SCNT models, UNC0638 treatment of donor cells (goat fetal fibroblasts, GFFs) reduced the elevated levels of H3K9 dimethylation (H3K9me2) commonly found in SCNT embryos. While this treatment did not improve overall in vitro developmental rates, UNC0638 did lead to beneficial changes in gene expression, particularly the upregulation of key developmental genes.^50^ These results suggest that UNC0638 may be able to restore normal epigenetic programming in SCNT embryos, enhancing their developmental potential without altering imprinted gene expression.

Collectively, these studies underscore the critical role of H3K9 methylation in regulating gene expression and cellular fate, with UNC0638 acting as a potent tool to modify these epigenetic marks. Whether used in the context of AML, where it can reprogram leukemia cell differentiation, or in SCNT, where it can correct epigenetic errors in cloned embryos, UNC0638 holds significant potential for therapeutic and reproductive applications. By targeting the enzymes responsible for H3K9 methylation, UNC0638 offers a unique approach to manipulate the epigenome in ways that could have broad implications for both disease treatment and developmental biology.^49^, ^50^

## 16 A‐366

A‐366 is a highly selective and potent inhibitor of the histone lysine methyltransferase G9a (EHMT2) and its closely related homolog GLP (EHMT1).^51^ It was developed through a unique diversity screening process, with optimization through the incorporation of a propyl‐pyrrolidine subunit that occupies the enzyme's lysine‐binding channel. A‐366 demonstrates impressive selectivity, inhibiting G9a and GLP with an IC50 of 3.3 nM, while showing more than 1000‐fold selectivity over 21 other methyltransferases.^52^

In cellular studies, A‐366 effectively reduces H3K9me2 levels, mirroring the activity of other G9a/GLP inhibitors, but with significantly lower cytotoxicity across a range of tumor cell lines. Notably, A‐366 has been found to exert less general toxicity compared to other known G9a/GLP inhibitors, suggesting a more favorable therapeutic profile. Furthermore, its selective inhibition of G9a/GLP has shed light on the enzyme's critical role in leukemia. Treatment of leukemia cell lines in vitro with A‐366 led to marked differentiation and morphological changes, indicating a potential therapeutic benefit in modifying the differentiation status of leukemia cells. In vivo studies using a flank xenograft leukemia model also demonstrated that A‐366 treatment resulted in significant tumor growth inhibition, correlating with a reduction in H3K9me2 levels, consistent with the observed in vitro effects.^53^ Overall, A‐366 is a novel and highly selective G9a/GLP inhibitor that has not only advanced our understanding of these enzymes in leukemia biology but also holds promise for future therapeutic strategies targeting G9a/GLP‐mediated epigenetic regulation in cancer.

## 17 (R,R)‐59

In 2020, researchers reported the development of the first selective and effective, (R,R)‐59, through stepwise structure‐guided optimization.^54^ SETDB1, which silences tumor suppressor genes and activate oncogenic AKT signaling,^20^ is overexpressed in various cancers.^21^ A unique feature of SETDB1 is its tandem Tudor domains (TTD), which recognize methylated lysines on histone H3, making it a promising target for cancer therapy. The development of (R,R)‐59 began with the identification of an initial hit compound (Cpd1), and through structural studies, (R,R)‐59 was optimized to become the first potent and selective small molecule inhibitor of SETDB1‐TTD, as determined by isothermal titration calorimetry (ITC). The compound's high potency was confirmed through a cocrystal structure of the (R,R)‐59‐TTD complex, revealing that it is an endogenous binder competitive inhibitor.^54^ Cellular target engagement was also demonstrated. Importantly, the enantiomer (S,S)‐59 showed no activity in the same assays, highlighting the specificity of (R,R)‐59. The crystal structure of the Cpd1‐TTD complex revealed that Cpd1 binds between the second and third Tudor domains, with its 1‐methyl‐3‐phenylpiperidine positioned within an aromatic cage formed by residues in the binding pocket. Additionally, the benzyloxy group in Cpd1 was found to induce a counterclockwise rotation of the indole ring, contributing to its enhanced binding affinity. These structural insights provided a foundation for the rational optimization of SETDB1‐TTD inhibitors, with (R,R)‐59 emerging as a potent and selective compound.^55^ While is initially found to be an inhibitor of SETDB1‐TTD, recent study surprisingly found that it induces SETDB1 activity to promote Akt K64 methylation.^55^ Future studies are needed to define the precise role of (R,R)‐59 in SETDB1's activity towards its various substrates.

## 18 CURRENT CLINICAL DRUGS AS SETDB1 ANTAGONISTS: MECHANISMS AND THERAPEUTIC POTENTIAL

**FIGURE 3:**
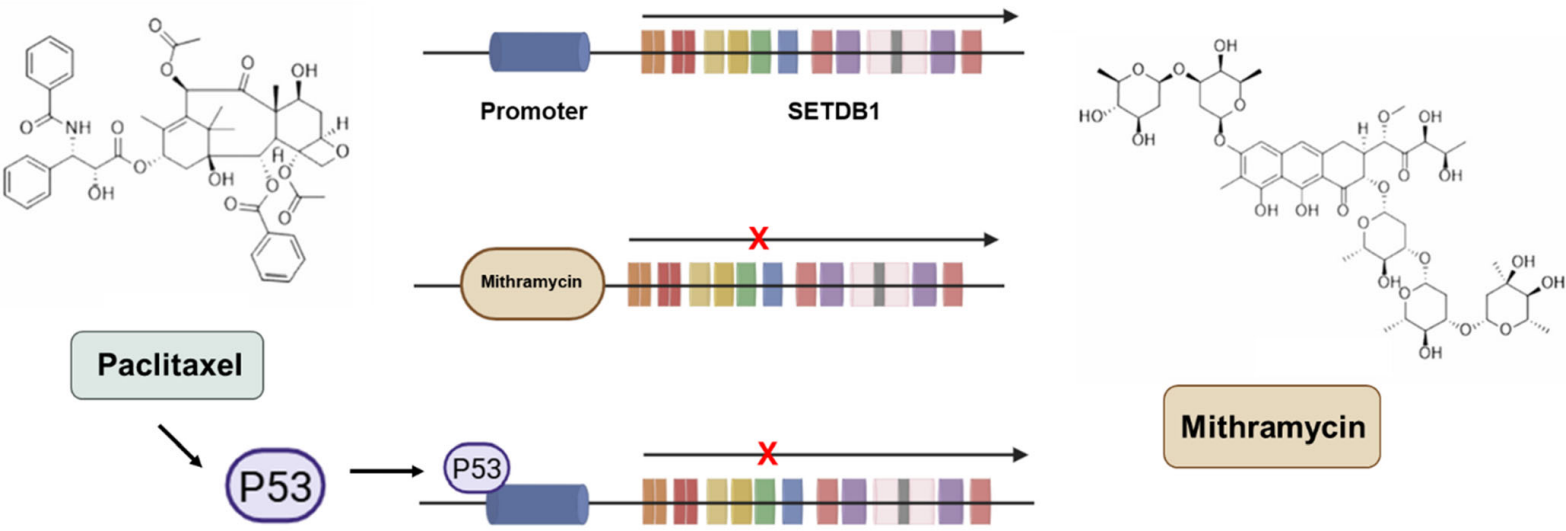
Current clinical drugs serve as SETDB1 antagonists. Mithramycin binds to the minor groove of GC‐rich DNA, displacing transcription factors like SP1 and SP3, thus leading to reduced SETDB1 expression. Paclitaxel treatment induces p53 expression, which suppresses SETDB1 promoter activity by binding directly to its proximal region leading to reduced SETDB1 expression.

The expression of SETDB1 is regulated through both transcriptional and post‐transcriptional mechanisms. Transcription factors, such as SP1, SP3, TCF4, and c‐Myc,^56^ have been identified as key regulators of SETDB1 promoter activity, influencing its expression at the transcriptional level. Although no specific SETDB1 inhibitor is currently available in clinical setting, several non‐specific inhibitors targeting histone methyltransferases (HMTs) have been explored, including mithramycin A, paclitaxel, and microRNAs like miR‐621 and miR‐381‐3p. These agents have shown potential in modulating SETDB1 activity, though the exact mechanisms remain poorly understood.^57^ Paclitaxel, mithramycin, and related agents are considered non‐specific inhibitors of SETDB1, but they offer insights into potential therapeutic strategies. In melanoma, a highly aggressive skin cancer, SETDB1 has been found to regulate key processes, such as the expression of cancer‐related secreted factors, melanocyte‐lineage differentiation, and metabolic pathways.^58^ Recent studies have shown that SETDB1 plays a significant role in melanoma pathogenesis. Inhibition of SETDB1 in melanoma cells has led to altered transcriptomic, morphological, and functional changes, supporting its potential as a therapeutic target.^58^ Treatment with non‐specific SETDB1 inhibitors, such as mithramycin A and its analog mithralog EC‐8042, resulted in strong anti‐melanoma effects, suppressing SETDB1 expression and enhancing the efficacy of mitogen‐activated protein kinase (MAPK) inhibitors. This suggests that SETDB1‐targeting strategies could be an effective addition to current melanoma treatments. Mithramycin, an antitumor antibiotic, binds to the minor groove of GC‐rich DNA, displacing transcription factors like SP1 and SP3 that are involved in SETDB1 activation.^59^ By interfering with these transcription factors, mithramycin downregulates SETDB1 expression and reduces H3K9 trimethylation, a hallmark of SETDB1 activity. Similarly, paclitaxel (PTX), a widely used chemotherapeutic agent, has been shown to downregulate SETDB1 expression in a p53‐dependent manner.^14^ PTX treatment induces p53 expression, which inhibits SETDB1 promoter activity by binding directly to its proximal region, increasing H3K9me3 occupancy, and repressing SETDB1 transcription.^60^ This mechanism has been observed in both melanoma and lung cancer cells, where PTX treatment reduces SETDB1 expression and enhances the effects of other treatments, such as MAPK inhibitors. Other chemotherapeutic agents, including doxorubicin, cisplatin, and 5‐fluorouracil, have also been reported to downregulate SETDB1 expression through transcriptional repression, indicating that SETDB1 inhibition may be a common response to anticancer therapies.^60^ Furthermore, the combination of PTX and SETDB1 inhibition leads to increased expression of FosB, a transcription factor linked to cell proliferation, suggesting that SETDB1‐mediated regulation of FosB may play a role in cancer progression during chemotherapy^61^ (Figure 3).

In conclusion, SETDB1 emerges as a critical regulator in melanoma and other cancers, and its inhibition through compounds like mithramycin and paclitaxel shows promise in enhancing the efficacy of existing therapies. These findings underscore the potential of SETDB1‐targeted strategies to improve cancer treatment outcomes, particularly in melanoma, where SETDB1 contributes to tumor progression and drug resistance. Further exploration of SETDB1 inhibitors in combination with other therapeutic agents could offer novel approaches to overcoming treatment resistance and improving patient prognosis.

## 19 CONCLUSION AND FUTURE DIRECTIONS

Cancer remains one of the deadliest diseases worldwide, with resistance to treatment being a key challenge in patient survival. Epigenetic alterations contribute significantly to therapy resistance by affecting the expression of oncogenes, silencing tumor suppressor genes, and altering signaling pathways involved in cancer progression, immune evasion, and drug resistance.^62^ Despite extensive research into epigenetic therapies, challenges, such as selectivity, off‐target effects, and potential inhibition of anticancer defense mechanisms persist. The need for developing safe, effective epigenetic drugs is crucial. SETDB1, a key epigenetic regulator, has emerged as a promising target in cancer treatment due to its role in silencing genes through H3K9me3 modifications and promoting oncogenic signaling through non‐histone protein methylation, contributing to tumor proliferation and resistance. SETDB1's dysregulation also affects immune cell development and function, with its inhibition enhancing immune responses and potentially improving cancer outcomes. However, SETDB1's complex regulatory network, involving cofactors like TRIM28 and the HUSH complex, presents challenges in developing selective therapies. Future research should focus on understanding SETDB1's mechanisms across different cancer types, developing SETDB1‐specific inhibitors, and mapping its role in immune regulation to refine therapeutic strategies. By addressing these issues, developing selective SETDB1 inhibitors could provide new avenues for effective cancer treatments with minimal adverse effects.

## CONFLICT OF INTEREST STATEMENT

H.K.L. is a consultant for Stablix, Inc. All other authors have declared that no competing interests exist.

## Data Availability

Data sharing is not applicable to this article as no new data were created or analyzed in this study.
